# Morphological Change to Birds over 120 Years Is Not Explained by Thermal Adaptation to Climate Change

**DOI:** 10.1371/journal.pone.0101927

**Published:** 2014-07-14

**Authors:** Volker Salewski, Karl-Heinz Siebenrock, Wesley M. Hochachka, Friederike Woog, Wolfgang Fiedler

**Affiliations:** 1 Michael-Otto-Institut im NABU, Bergenhusen, Germany; 2 Max-Planck-Institute for Ornithology, Department of Migration and Immuno-ecology, Radolfzell, Germany; 3 Lab of Ornithology, Cornell University, Ithaca, New York, United States of America; 4 Staatliches Museum für Naturkunde Stuttgart, Stuttgart, Germany; Institute of Ecology, Germany

## Abstract

Changes in morphology have been postulated as one of the responses of animals to global warming, with increasing ambient temperatures leading to decreasing body size. However, the results of previous studies are inconsistent. Problems related to the analyses of trends in body size may be related to the short-term nature of data sets, to the selection of surrogates for body size, to the appropriate models for data analyses, and to the interpretation as morphology may change in response to ecological drivers other than climate and irrespective of size. Using generalized additive models, we analysed trends in three morphological traits of 4529 specimens of eleven bird species collected between 1889 and 2010 in southern Germany and adjacent areas. Changes and trends in morphology over time were not consistent when all species and traits were considered. Six of the eleven species displayed a significant association of tarsus length with time but the direction of the association varied. Wing length decreased in the majority of species but there were few significant trends in wing pointedness. Few of the traits were significantly associated with mean ambient temperatures. We argue that although there are significant changes in morphology over time there is no consistent trend for decreasing body size and therefore no support for the hypothesis of decreasing body size because of climate change. Non-consistent trends of change in surrogates for size within species indicate that fluctuations are influenced by factors other than temperature, and that not all surrogates may represent size appropriately. Future analyses should carefully select measures of body size and consider alternative hypotheses for change.

## Introduction

Changes in morphology, and in particular a decline in body size, are expected to be one of many responses of animals to current rapid global warming [1], [2]. Fluctuations in the body size of mammals during the Holocene, sometimes with shifts of up to 25% within a century, are correlated with climatic change [3]–[5]. Animals introduced to new environments changed morphology and size within a few decades [6]–[8], and morphology can change rapidly when driven by selection [9], [10]. Previous authors [2], [11], [12] have suggested that changes in body size follow Bergmann's prediction [13] that endotherms should be smaller in warmer climates because of thermoregulatory needs. However, Bergmann [13] himself discussed species-specific factors (e.g. body insulation, habitat use, behaviour, nutrition) that may mask the predicted size trend (see also [14], [15]). Therefore, the assumption of unidirectional changes in morphology as a response to a single environmental factor (temperature) across a wide range of species with different ecologies may be questioned, and a review of recent studies examining trends in body size produced conflicting results [16]. Birds and mammals have shown trends consistent with [12], [17], [18], and contrary to [19], [20], the prediction of decreasing body size with warmer temperatures and lower latitudes, or no clear trend [21]–[23]. Some studies of multiple species experiencing the same climate regime have found opposing directions of change among them [11], [16], [23], [24].

There are several potential caveats to the analyses and interpretation of body size trends in animals [25]. First, selecting an appropriate measure to represent body size may be problematic. Examples are the use of the size of teeth in studies of quaternary mammals or the use of body mass in studies of body size of birds [24], [26]. Ecological factors such as habitat structure, food availability or competition within a community may influence the size of organs such as teeth, bills or limbs independently of body size [24], [27]–[29].

A second caveat is related to the structure and interpretation of analyses. In birds, for example, body mass or wing length is frequently used as surrogates for body size [30]. However, body mass varies with season, time of the day, temperature and moult status [23], [26], and wing length may vary with age, sex and the age of the feathers [31]. Ignoring such associations may lead to spurious results. Furthermore, the increase in temperature associated with recent climate change has not been linear; during the past hundred years there have been periods of both rising and falling temperature, with the recent steep increase of global temperatures starting in the early 1970s [32]. Therefore, potential adjustments of size to the prevailing temperature regime could lead to fluctuations rather than linear trends. However, many studies using long-term data sets have used linear models or compared values of one time period with another [11], [12], [17] even though forcing linear regressions through non-linear trends is problematic.

The third caveat is that the time span of the data set needs to be long enough to distinguish between long-term trends and microevolutionary adaptive change such as may be caused by year-to-year variation of local weather [33]–[35]. Even under the scenario of recent climate change, local fluctuations in weather conditions may trigger phenotypic responses and thus better explain morphological change in short-term data sets [35]. However, analyses of body-size trends using data going back for more than 50 years are scarce [11], [12].

Here, we present an analysis of morphological change based on museum specimens of eleven bird species collected in southern Germany and adjacent areas between 1889 and 2010. We examine variation over time in three aspects of morphology (tarsus length, wing length and the Kipp-index of wing pointedness), using additive models that allow for non-linear change. We additionally control for the potential effects of month of collection, sex and age of the individuals. Thus, our study was designed to address the caveats noted above. With respect to the assumption of generally decreasing body sizes in times of global warming [2], the goals of our study are: 1) to determine whether there has been significant long-term variation in morphology over time across all species; 2) to identify differences among species in morphological trends that may be associated with ecological drivers other than temperature; and 3) to identify associations between the observed trends in morphology and changes in climate in the study area.

## Materials and Methods

### Study area

The morphology of birds shows intraspecific latitudinal variation which is mostly correlated with migratory behaviour [36], [37]. Therefore, we restricted our study to specimens that were collected in southern Germany and adjacent areas in Switzerland and Austria between about 47.0°N and 50.8°N and between about 6.9°E and 14.3°E. The great majority of the specimens used (75%) were collected in the German federal states of Baden-Württemberg and Bavaria between 47.27° and 50.57°N and between 7.5°E and 13.8°E.

### Selection of study species

We selected a range of species that represent different migration strategies because non-migratory bird species follow Bergmann's rule more strongly than migrants [38]. The migration categories considered were: long-distance migrants from the study area to sub-Saharan Africa, short-distance or partial migrants in which either the entire or a proportion of the population migrates to the Mediterranean, and residents. Initially, we selected six species out of each migration category according to the catalogue of the Rosenstein Museum in Stuttgart, but after the visits to the first two and largest collections (Stuttgart, Munich), we discovered that we could not expect to get sufficient specimens for many species, especially for long-distance migrants. For this reason we restricted our analyses to eleven of our initial 18 target species. Of the eleven species, we measured 4529 specimens collected between 1889 and 2010 (Table S1). Some sample sizes for specific analyses may be lower than the totals of measured specimens because it was not possible to measure all morphological variables in all specimens, and specimens were excluded when they did not meet our criteria for certain analyses (see below).

### Measurements

Measurements were taken in collections which are listed in the Acknowledgements.

It has been debated which measurements represent the size of birds most adequately [26], [30], [39], [40]. Several studies proposed tarsus length as the preferred single proxy for body size [39]–[41]. Gosler et al. [30] described wing length as the best single linear proxy of body size (but see [39], [41], [42]). However, wing length is also related to migration and habitat use [43], [44]. Wing pointedness is related to the efficiency of long-distance flight, with migrants having more pointed wings [36], [37], [45], [46].We considered tarsus length, wing length and the Kipp-index of wing pointedness in parallel to appraise whether trends were consistent across all traits and whether alternative explanations suggest some of these traits might not be good surrogates for body size. With respect to the expectation of decreasing body size [2] and reduced migratory activity [47] as a response to the current global warming, the three measurements are expected to decrease during the study period and especially during the last four decades.

Wing length was measured from the wrist to the tip with a butted ruler (Wmax [48]), and the primary projection with a piece of laminated millimetre-gridded paper with a precision of 0.5 mm. The tarsus was measured by taking the distance between the back of the intertarsal joint and the lower front edge of the last undivided scale before the toes diverge (Tar 2 in [48]). The length was marked using the tip of dividers (pair of compasses) and the length read from them on millimetre-gridded paper under a magnifying lamp with a precision of 0.1 mm. The Kipp-index [49], hereafter “Kipp”, is calculated as the percentage of the primary projection (distance from the tip of the first secondary feather to the tip of the longest primary feather) of the wing length (Kipp  =  primary projection/wing length*100).

All measurements were taken by K.-H. Siebenrock, thereby avoiding inter-measurer variation [30], [50]. We analysed within-observer consistency by blindly repeating the measurements of 98 blackbirds *Turdus merula* (97 for tarsus) in 2012 that were first measured in 2007. The mean of the repeated values differed by <0.01 mm for tarsus length, 0.02 mm for wing length and 0.04 mm for the primary projection. Analysing the data according to Lessells & Boag [51], repeatabilities were 50%, 97% and 84% for tarsus length, wing length and primary projection respectively. The repeatability for tarsus length was surprisingly low in contrast to other studies [52]–[54], which is probably due to the sometimes problematic assignment of a reference point for measurements of museum specimens [55]. Nevertheless, these inconsistencies in tarsus measurements did not vary with year of collection of specimens (hereafter: year); a linear model with the differences between two measurements as dependent and year as the independent variable revealed an equal distribution of differences over time (Figure S1). Therefore, we consider that any trends over time in our data are not influenced by low repeatability although confidence intervals around estimates of temporal trends may be higher for tarsus length.

### Data analyses

#### Generalized Additive Models

We used generalized additive models (GAMs), implemented in the package mgcv of the programme R 2.11.1 [56], to associate morphological variables with year and temperature. GAMs allow for arbitrary variation in the target variables through time [57], [58] that potentially describe fluctuations of morphological characters better than linear models, while still approximating linear trends if these are biologically real. In order to avoid possible errors in interpretation due to biased availability of specimens by age or sex cohorts, or due to the presence of individuals that only migrated to or through the study area, we performed a two-step analysis for every species and morphological trait. In the first step, we considered only specimens with known sex and age (before the first primary moult or afterwards) according to Svensson [55], Jenni & Winkler [59] or the label on the specimen, as well as specimens with known month of collection. We then used ANOVA to test for potential associations of sex, age or month of collection with tarsus length, wing length or Kipp. In the second step, we fitted GAMs to the data with either year or temperature as a smoothing term and the variables that were significant in the first step as fixed factors. Specimens for which information was not available for inclusion at the first step were included at the second step if the relevant variable was not significant at the first, thus considerably increasing our sample sizes for the GAMs.

#### Climate data

**Figure 1:**
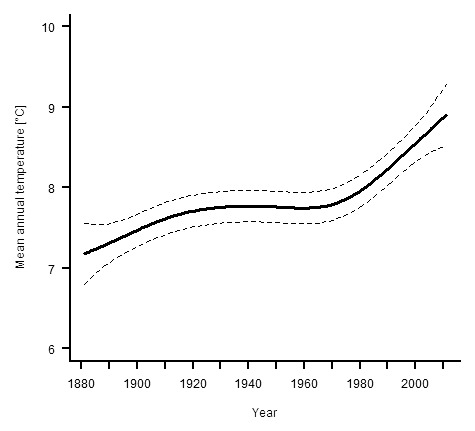
Mean annual temperature in southern Germany between 1881 and 2011. Solid line – regression spline fit from the GAM; dashed lines – 95% confidence intervals.

Mean annual temperatures between 1881 and 2011 for the German states of Länder Baden-Wuerttemberg and Bavaria were obtained from the Deutscher Wetterdienst (www.dwd.de). A GAM revealed that the smooth term year was significantly associated with temperature in the study area (F_3.72, 126.28_ = 13.58, p<0.001, adjusted R^2^ = 0.323). During the study period, there was a general non-linear trend for increasing temperatures (Figure 1). Between 1881 and about 1940, temperatures generally increased. From then until about 1970, temperatures decreasing slightly, followed by a steep increase in temperatures to the present day that is consistent with recent global warming [32].

Conditions during certain seasons may have a greater influence on survival compared to other seasons [60]. Therefore, morphological adaptations to conditions during a crucial period within the annual cycle may not be detected when using temperature means over the entire annual cycle. However, linear regressions revealed that mean temperatures during seasons were significantly correlated with the temperature of the respective years: spring (March-May; n = 132, _F1,128_ = 130.4, adjusted R^2^ = 0.50, p = <0.001), summer (June-August; n = 132, _F1,128_ = 49.62, adjusted R^2^ = 0.27, p = <0.001), autumn (September-November; n = 132, _F1,128_ = 27.15, adjusted R^2^ = 0.17, p = <0.001) and winter (December-February; n = 132, _F1,128_ = 59.93, adjusted R^2^ = 0.31, p = <0.001). Therefore, seasonal trends in temperature were not considered further.

Temperature in the year of collection may not be the most biologically-appropriate year to correlate with morphological characters. Many specimens have been collected early in the year and feathers grew in the previous year. Tarsi are fully grown when birds fledge [61] and thus temperature in subsequent years has no direct influence on the tarsus length of older individuals. Further, phenotypic expression of morphological characters may be more influenced by selective pressures acting in previous years. Therefore, we examined correlations between morphology and running means of temperature for the year of collection and the four preceding years. All species considered in this study have a generation length of less than 3.5 years [62]. Therefore, our approach includes temperatures experienced when the respective traits developed as well as temperatures causing potential selective pressures on one or two previous generations of most individuals considered in this study. Hence, temperatures that may cause phenotypic responses as well as microevolutionary adaptations are included.

To test whether temperature explained fluctuations in morphology better than a time trend alone, we fitted similar GAMs to the same data as described above, but with temperature instead of year as the smooth term. We compared pairs of GAMs (temperature, year) for each morphological trait and species with a likelihood ratio test. The accepted significance level was p≤0.05.

## Results

The first step of our analyses revealed that month of collection was significantly associated with variation in measurements in six cases (tarsus length – 1 species, wing length – 3 species, Kipp – 2 species; Table 1). Sex was significantly associated with the morphological traits in 13 (tarsus length – 4 species, wing length – 8 species, Kipp – 1 species; Table 1) and age in 16 cases (wing length – 8 species, Kipp – 8 species; Table 1) respectively.

**Table 1:** Associations of morphological traits with the age, sex and month of collection of specimens in 11 bird species.

Species	Morphological trait (n)	Age			Sex			Month		
		df	F	p	df	F	p	df	F	p
Great Spotted Woodpecker	Tarsus length (266)	1, 163	0.097	0.756	1, 163	1.925	0.167	11, 163	1.344	0.205
	Wing length (241)	**1, 141**	**11.909**	**0.001**	1, 141	0.620	0.432	11, 141	1.386	0.186
	Kipp (233)	**1, 134**	**7.664**	**0.006**	1, 134	2.556	0.112	**11, 134**	**1.941**	**0.039**
Robin	Tarsus length (288)	1, 184	1.108	0.294	1, 184	0.149	0.700	11, 184	0.928	0.515
	Wing length (286)	1, 182	1.890	0.171	**1, 182**	**20.614**	**<0.001**	11, 182	0.494	0.905
	Kipp (284)	1, 180	1.412	0.236	1, 180	3.322	0.070	11, 180	1.093	0.370
Blackbird	Tarsus length (459)	1, 345	<0.001	0.998	1, 345	3.157	0.076	11, 345	1.301	0.222
	Wing length (427)	**1, 313**	**63.757**	**<0.001**	**1, 313**	**186.986**	**<0.001**	**11, 313**	**1.846**	**0.046**
	Kipp (426)	1, 312	3.395	0.066	1, 312	0.055	0.815	11, 312	0.663	0.773
Song Thrush	Tarsus length (130)	1, 56	0.699	0.407	**1, 56**	**7.928**	**0.007**	**11, 56**	**2.210**	**0.026**
	Wing length (125)	1, 52	0.702	0.406	**1, 52**	**7.462**	**0.009**	11, 52	1.456	0.177
	Kipp (125)	1, 52	1.300	0.259	1, 52	0.996	0.323	11, 52	0.771	0.666
Great Tit	Tarsus length (444)	1, 341	1.392	0.239	**1, 341**	**19.203**	**<0.001**	11, 341	0.780	0.660
	Wing length (435)	**1, 331**	**69.164**	**<0.001**	**1, 331**	**148.696**	**<0.001**	11, 331	1.175	0.303
	Kipp (433)	**1, 329**	**21.871**	**<0.001**	1, 329	0.569	0.451	**11, 329**	**1.890**	**0.040**
Blackcap	Tarsus length (123)	1, 49	0.319	0.575	1, 49	0.138	0.712	7, 49	0.758	0.625
	Wing length (119)	1, 47	1.186	0.282	1, 47	0.029	0.866	7, 47	0.955	0.475
	Kipp (119)	**1, 47**	**10.019**	**0.003**	1, 47	0.008	0.928	7, 47	0.877	0.531
Starling	Tarsus length (361)	1, 270	0.380	0.538	**1, 270**	**5.673**	**0.018**	10, 270	0.402	0.945
	Wing length (316)	**1, 225**	**73.273**	**<0.001**	**1, 225**	**39.062**	**<0.001**	10, 225	1.433	0.167
	Kipp (268)	**1, 179**	**11.504**	**0.001**	1, 179	0.069	0.793	10, 179	1.729	0.077
Greenfinch	Tarsus length (255)	1, 156	0.307	0.580	1, 156	0.012	0.912	11, 156	1.107	0.359
	Wing length (243)	**1, 146**	**11.343**	**0.001**	**1, 146**	**30.467**	**<0.001**	11, 146	1.090	0.374
	Kipp (241)	**1, 144**	**14.397**	**<0.001**	1, 144	3.240	0.074	11, 144	1.547	0.121
Bullfinch	Tarsus length (450)	1, 352	0.095	0.758	1, 352	0.025	0.875	11, 352	0.707	0.732
	Wing length (445)	**1, 348**	**16.484**	**<0.001**	**1, 348**	**22.252**	**<0.001**	**11, 348**	**2.084**	**0.021**
	Kipp (440)	**1, 343**	**95.798**	**<0.001**	**1, 343**	**5.700**	**0.018**	11, 343	1.317	0.213
House Sparrow	Tarsus length (642)	1, 341	1.392	0.239	**1, 341**	**19.203**	**<0.001**	11, 341	0.780	0.660
	Wing length (599)	**1, 489**	**62.334**	**<0.001**	**1, 489**	**226.467**	**<0.001**	**11, 489**	**2.166**	**0.015**
	Kipp (578)	**1, 144**	**14.397**	**<0.001**	1, 144	3.240	0.074	11, 144	1.547	0.121
Red-backed Shrike	Tarsus length (239)	1, 164	0.138	0.711	1, 164	2.359	0.127	5, 164	1.939	0.090
	Wing length (225)	**1, 153**	**7.703**	**0.006**	1, 153	0.666	0.416	5, 153	1.244	0.291
	Kipp (224)	**1, 152**	**13.800**	**<0.001**	1, 152	0.725	0.396	5, 152	1.433	0.215

Shown are the degrees of freedom (df) as well as F and p values of ANOVA with the morphological trait as the dependent factor and age, sex and month of collection as explanatory variables. Bold: p<0.05.

**Figure 2:**
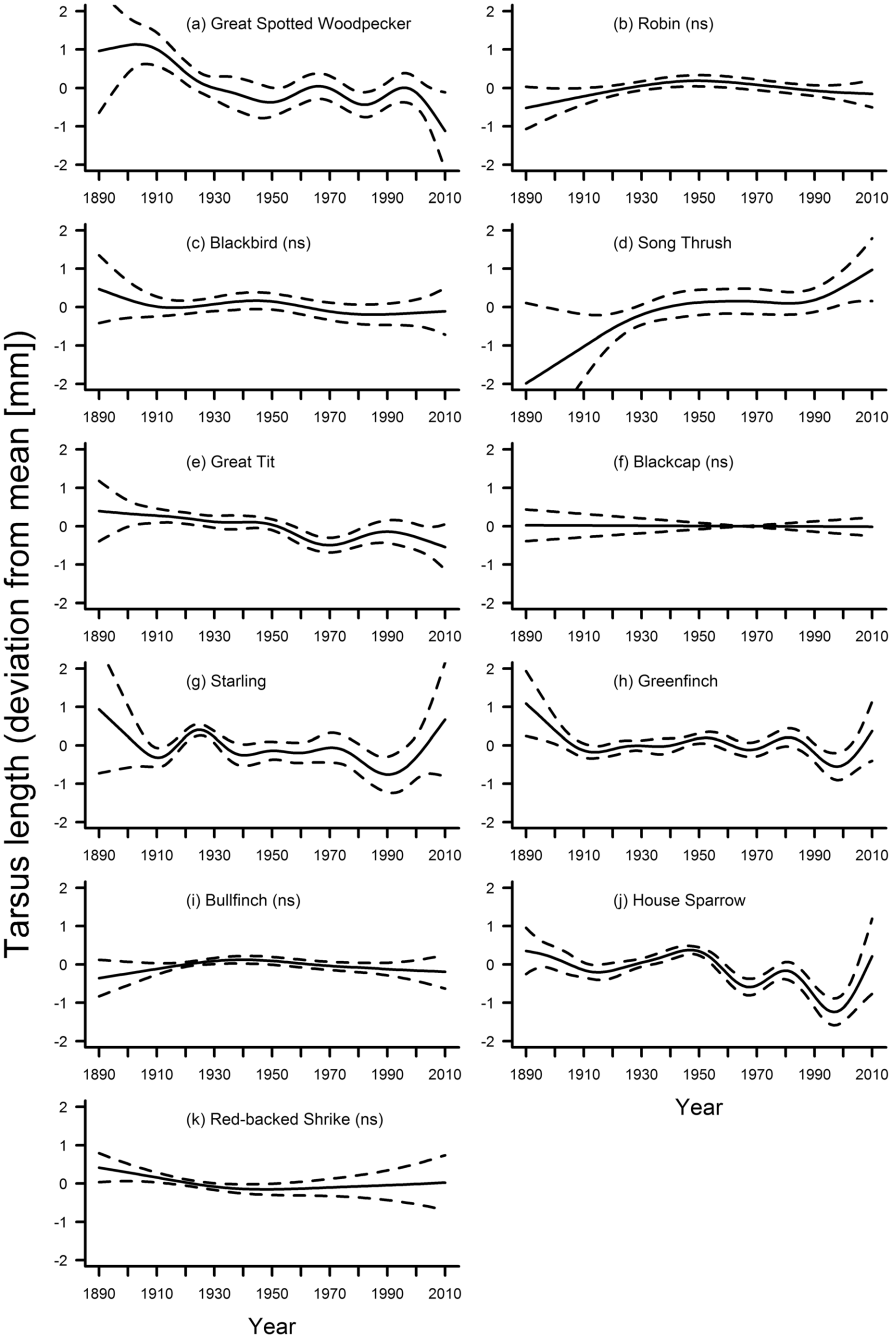
Fluctuations in tarsus length of eleven bird species in southern Germany between 1889 and 2010. Solid line – regression spline fit from the GAM; dashed lines – 95% confidence intervals. See methods for details. A p-value of >0.05 is indicated with (ns) after the species name, see table 2 for exact p-values.

**Figure 3:**
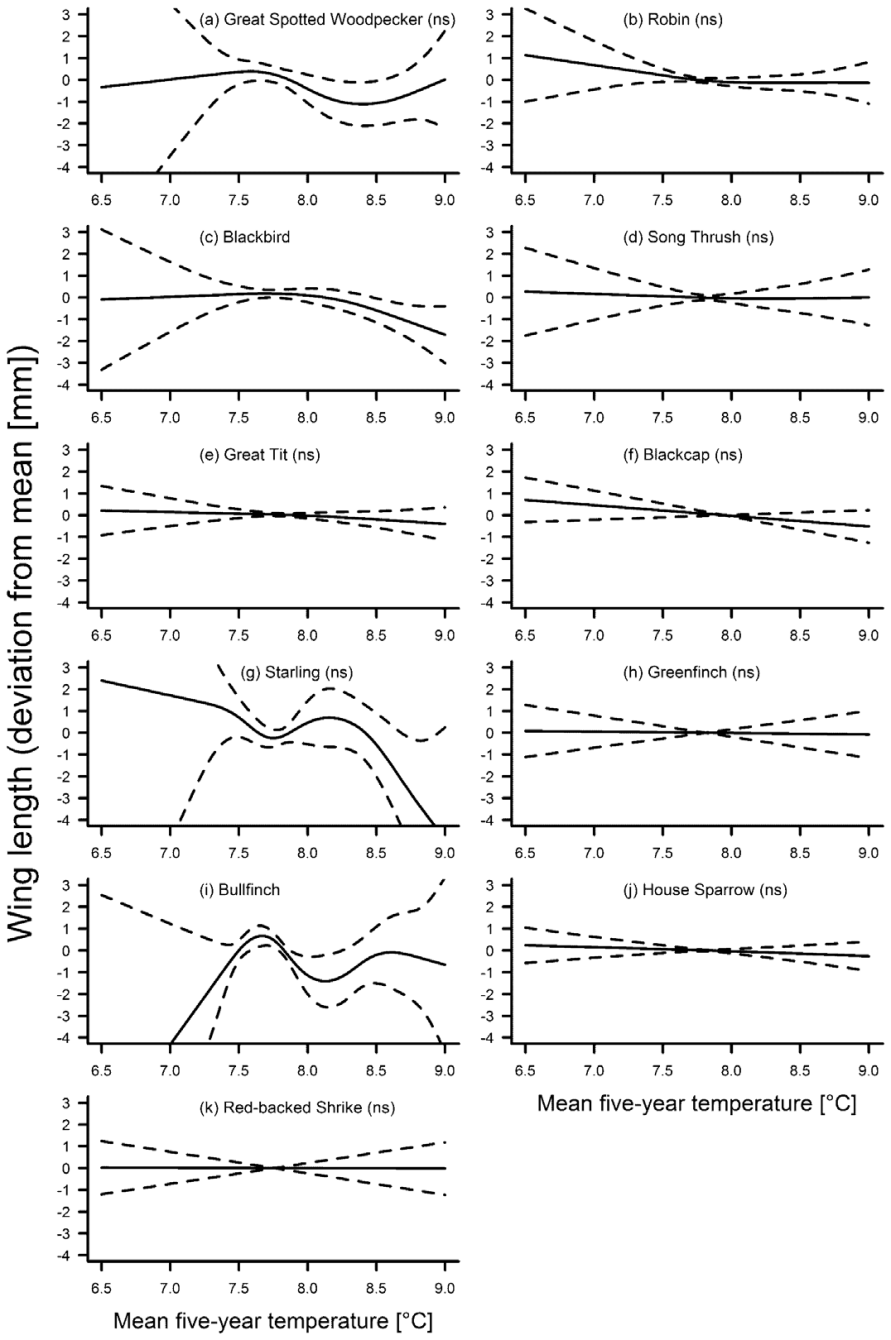
Fluctuations in wing length of eleven bird species in association with the five-year mean annual temperature. Solid line – regression spline fit from the GAM; dashed lines – 95% confidence intervals. See methods for details. A p-value of >0.05 is indicated with (ns) after the species name, see table 3 for exact p-values.

**Figure 4:**
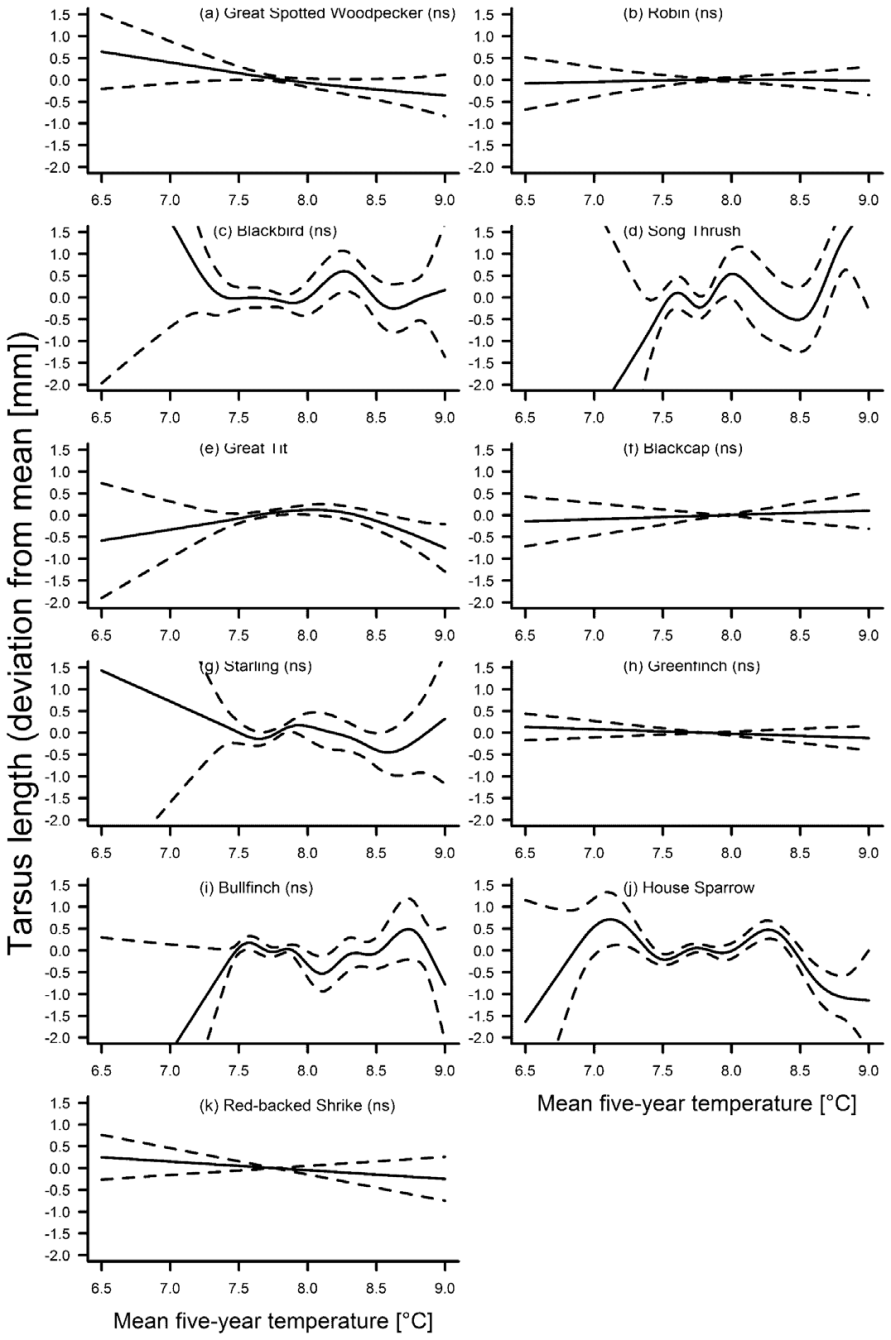
Fluctuations in tarsus length of eleven bird species in association with the five-year mean annual temperature. Solid line – regression spline fit from the GAM; dashed lines – 95% confidence intervals. See methods for details. A p-value of >0.05 is indicated with (ns) after the species name, see table 3 for exact p-values.

The second step of our analyses revealed that tarsus length varied significantly with the smooth term year in six species (Table 2). Of these, tarsus length in the song thrush *Turdus philomelos* increased with an especially pronounced trend during the last two decades (Figure 2d). In the great-spotted woodpecker (Figure 2a) and the great tit *Parus major* (Figure 2e), the overall trend for tarsus length was decreasing, whereas three species (starling *Sturnus vulgaris*, greenfinch *Chloris chloris*, house sparrow *Passer domesticus*, Figure 2g,h,j) showed fluctuating tarsus lengths without a clear trend throughout the study period. The similarity of trends from ca. 1940 to 2010 in these last three species, and especially the pronounced increasing trend during the last decades of the study period (Figure 2g,h,j), is remarkable and was also apparent in the song thrush. In only three species was tarsus length significantly associated with the five-year mean of temperature (Table 3), but there was no clear general trend in tarsus length in association with temperature in these three species. However, in the great tit and house sparrow, tarsus length decreased distinctly when mean temperatures were relatively high (Figure 3e,j), though in the song thrush the opposite was the case (Figure 4d). In the song thrush, the GAM including temperature as a smooth term explained significantly more variation in tarsus length than the GAM including year as the smooth term. In the great tit and the house sparrow, the opposite was the case (Table 4).

**Table 2:** Association of three morphological variables for 11 bird species with the smooth term “year” in a GAM.

Species	Morphological trait (n)	df	F	p	Adjusted R^2^
Great Spotted Woodpecker	Tarsus length (311)	7.2, 302.8	4.327	**<0.001**	0.097
	Wing length (254)	6.7, 245.3	2.961	**0.004**	0.186
	Kipp (245)	1,5, 230.5	0.472	0.607	0.146
Robin	Tarsus length (376)	2.7, 372.3	2.170	0.082	0.019
	Wing length (298)	1.0, 295.0	1.772	0.184	0.096
	Kipp (371)	2.1, 367.9	1.531	0.210	0.008
Blackbird	Tarsus length (513)	4.0, 508.0	1.070	0.376	0.005
	Wing length (427)	1.7, 411.3	9.868	**<0.001**	0.416
	Kipp (468)	3.8, 463.2	2.718	**0.022**	0.024
Song Thrush	Tarsus length (211)	3.4, 194.6	3.005	**0.017**	0.128
	Wing length (197)	7.8, 187.2	2.842	**0.004**	0.197
	Kipp (257)	3.9, 252.1	0.951	0.447	0.010
Great Tit	Tarsus length (447)	6.2, 438.1	5.698	**<0.001**	0.119
	Wing length (438)	1.0, 434.0	4.663	**0.031**	0.356
	Kipp (438)	1.3, 423.7	0.547	0.535	0.085
Blackcap	Tarsus length (192)	1.0, 190.0	0.013	0.909	<0.001
	Wing length (186)	1.7, 183.3	4.886	**0.007**	0.051
	Kipp (126)	2.8, 121.2	3.191	**0.020**	0.089
Starling	Tarsus length (369)	7.9, 359.1	4.440	**<0.001**	0.099
	Wing length (321)	5.5, 312.5	1.632	0.130	0.407
	Kipp (308)	1.5, 304.5	1.540	0.217	0.126
Greenfinch	Tarsus length (510)	8.5, 500.5	2.916	**0.002**	0.034
	Wing length (244)	1.0, 240.0	0.665	0.416	0.163
	Kipp (242)	1.0, 239.0	0.039	0.844	0.124
Bullfinch	Tarsus length (508)	2.8, 504.2	2.031	0.097	0.013
	Wing length (445)	1.0, 430.0	4.656	**0.032**	0.091
	Kipp (447)	1.0, 443.0	0.833	0.362	0.215
House Sparrow	Tarsus length (662)	8.7, 651.3	12.940	**<0.001**	0.145
	Wing length (599)	7.4, 577.6	7.081	**<0.001**	0.448
	Kipp (594)	1.0, 591.0	1.092	0.297	<0.001
Red-backed Shrike	Tarsus length (264)	2.2, 260.8	2.464	0.068	0.025
	Wing length (245)	1.0, 242.0	2.030	0.156	0.062
	Kipp (244)	1.0, 241.0	8.366	**0.004**	0.107

Shown are the degrees of freedom (df), F and p values of the smooth term as well as the adjusted R^2^ of the models. Bold: p<0.05. See methods for details.

**Table 3:** Association of three morphological variables for 11 bird species with the smooth term “temperature” in a GAM.

Species	Morphological trait (n)	df	F	p	Adjusted R^2^
Great Spotted Woodpecker	Tarsus length (311)	1.3, 308.7	2.088	0.138	0.010
	Wing length (254)	2.8, 249.2	1.757	0.147	0.134
	Kipp (245)	1.0, 231.0	2.559	0.111	0.152
Robin	Tarsus length (376)	1.2, 373.8	0.047	0.889	<0.001
	Wing length (298)	1.7, 294.3	0.833	0.442	0.097
	Kipp (371)	1.3, 368.7	6.380	**0.004**	0.028
Blackbird	Tarsus length (513)	6.4, 505.6	1.171	0.316	0.011
	Wing length (427)	1.9, 411.1	3.641	**0.020**	0.399
	Kipp (468)	5.1, 461.9	2.926	**0.007**	0.033
Song Thrush	Tarsus length (211)	6.5, 191.5	2.991	**0.004**	0.162
	Wing length (197)	1.1, 193.9	0.017	0.934	0.113
	Kipp (257)	1.7, 254.3	1.642	0.194	0.010
Great Tit	Tarsus length (447)	2.5, 442.5	3.742	**0.010**	0.067
	Wing length (438)	1.2, 433.8	0.475	0.556	0.351
	Kipp (438)	7.0, 418.0	2.332	**0.018**	0.114
Blackcap	Tarsus length (192)	1.0, 190.0	0.253	0.615	<0.001
	Wing length (186)	1.0, 184.0	1.892	0.171	0.005
	Kipp (126)	2.1, 122.0	1.912	0.140	0.044
Starling	Tarsus length (369)	4.9, 362.1	1.508	0.176	0.036
	Wing length (321)	3.8, 315.2	1.691	0.140	0.117
	Kipp (308)	1.0, 305.0	0.128	0.720	0.119
Greenfinch	Tarsus length (510)	1.0, 508.0	0.755	0.385	<0.001
	Wing length (244)	1.0, 240.0	0.020	0.888	0.161
	Kipp (242)	1.0, 239.0	1.237	0.267	0.128
Bullfinch	Tarsus length (508)	8.0, 499.0	1.469	0.159	0.017
	Wing length (445)	4.5, 426.5	2.578	**0.022**	0.110
	Kipp (447)	1.0, 443.0	0.003	0.954	0.213
House Sparrow	Tarsus length (662)	7.8, 652.2	6.170	**<0.001**	0.069
	Wing length (599)	1.1, 583.9	0.443	0.525	0.397
	Kipp (594)	1.0, 591.0	0.025	0.874	<0.001
Red-backed Shrike	Tarsus length (264)	1.0, 262.0	0.957	0.329	<0.001
	Wing length (245)	1.0, 242.0	0.001	0.972	0.054
	Kipp (244)	7.3, 234.7	2.208	**0.026**	0.133

Shown are the degrees of freedom (df), F and p values of the smooth term as well as the adjusted R^2^ of the models. Bold: p<0.05. See methods for details.

**Table 4:** Comparisons of models with alternate smooth terms (temperature – year) for each species and morphological trait.

Species	Morphological trait	Δdf	ΔDeviance	p
Great Spotted Woodpecker	Tarsus length	−6.0	−52.3	**<0.001**
	Wing length	−3.9	−204.2	**<0.001**
	Kipp	−0.5	2.8	-
Robin	Tarsus length	−1.6	−9.3	**0.004**
	Wing length	0.7	3.3	0.204
	Kipp	−0.8	<0.1	-
Blackbird	Tarsus length	2.4	10.7	0.101
	Wing length	0.2	−96.4	-
	Kipp	1.3	9.4	**0.030**
Song Thrush	Tarsus length	3.1	19.1	**0.013**
	Wing length	−6.6	−172.7	**<0.001**
	Kipp	−2.2	−3.5	0.373
Great Tit	Tarsus length	−3.7	−22.0	**<0.001**
	Wing length	0.2	−12.4	-
	Kipp	5.7	31.7	**0.003**
Blackcap	Tarsus length	−0.0	0.3	-
	Wing length	−0.7	−35.4	**0.001**
	Kipp	−0.8	−14.0	**0.006**
Starling	Tarsus length	−3.0	−23.8	**<0.001**
	Wing length	−2.7	−1496.7	**<0.001**
	Kipp	−0.5	−5.8	**0.036**
Greenfinch	Tarsus length	−7.5	−14.5	**0.001**
	Wing length	0.0	−3.0	-
	Kipp	−0.0	3.2	-
Bullfinch	Tarsus length	5.1	3.6	0.221
	Wing length	3.5	96.2	**0.009**
	Kipp	−0.0	−1.4	**<0.001**
House Sparrow	Tarsus length	−0.9	−38.0	**<0.001**
	Wing length	−6.3	−244.2	**<0.001**
	Kipp	0.0	−2.1	-
Red-backed Shrike	Tarsus length	−1.2	−6.5	**0.007**
	Wing length	0.0	−9.3	-
	Kipp	6.3	21.4	**0.042**

Δdf and ΔDeviances describe the differences between paired models (GAMs), with negative values indicating that the model including “year” as a smooth term explains more variance than the model including “temperature” as the smooth term. P-values were calculated from F-tests, whose degrees of freedom were Δdf. For contrasts in which the degrees of freedom differed by less than 1, no statistical comparisons were possible.

**Figure 5:**
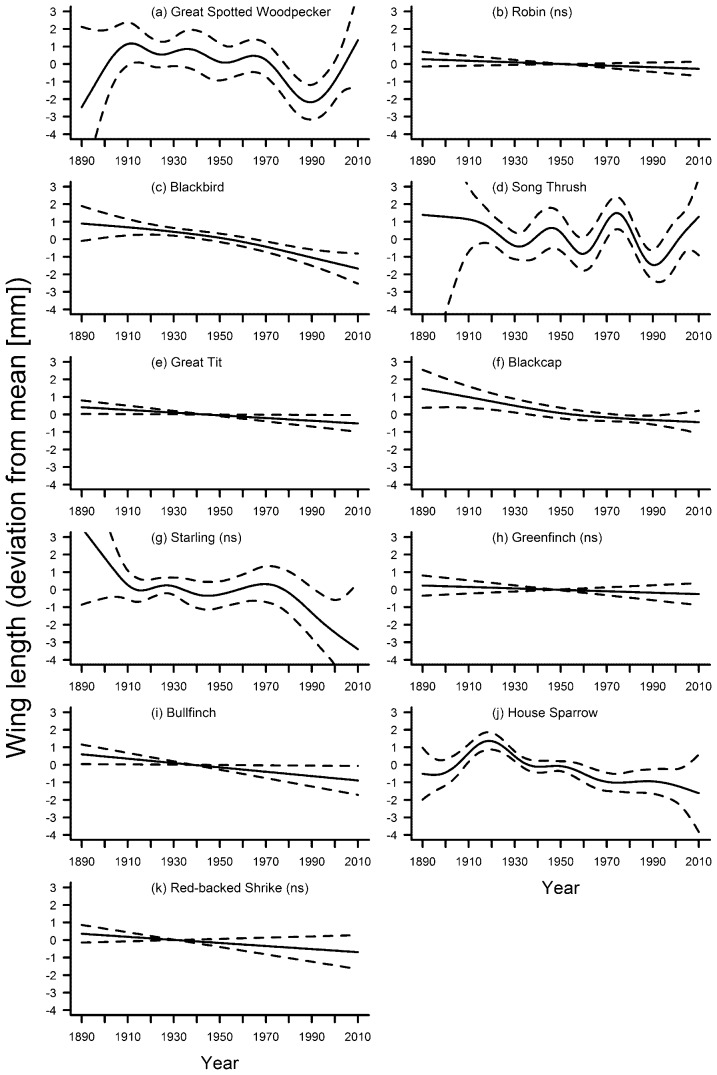
Fluctuations in wing length of eleven bird species in southern Germany between 1889 and 2010. Solid line – regression spline fit from the GAM; dashed lines – 95% confidence intervals. See methods for details. A p-value of >0.05 is indicated with (ns) after the species name, see table 2 for exact p-values.

The smooth term year was a significant predictor of wing length in seven species (Table 2). Amongst these species, there was no particular trend over the entire study period in the great-spotted woodpecker and the song thrush, both showing a distinct increase in wing length during the most recent decades (Figure 5a,d). Five species (blackbird, great tit, blackcap *Sylvia atricapilla*, bullfinch *Pyrrhula pyrrhula*, house sparrow; Figure 5c,e,f,i,j) showed a significant overall decrease in wing length. The starling also showed a distinct decrease in wing length since the 1970s (Figure 5g), but the overall association of wing length with year was not significant (Table 2). The linearly decreasing wing lengths of the robin *Erithacus rubecula*, the greenfinch and the red-backed shrike were also not significant (Figure 5b,h,k). In only two species was wing length significantly associated with the five-year mean of temperature (Table 3). Wing length decreased with higher temperatures in the blackbird (Figure 3c) but there was no distinct trend of wing length with temperature in the bullfinch (Figure 3i). In the blackbird, the GAM including year as a smooth term explained more variation of the data compared to the GAM including temperature as the smooth term. In the bullfinch, the opposite was the case (Table 4).

**Figure 6:**
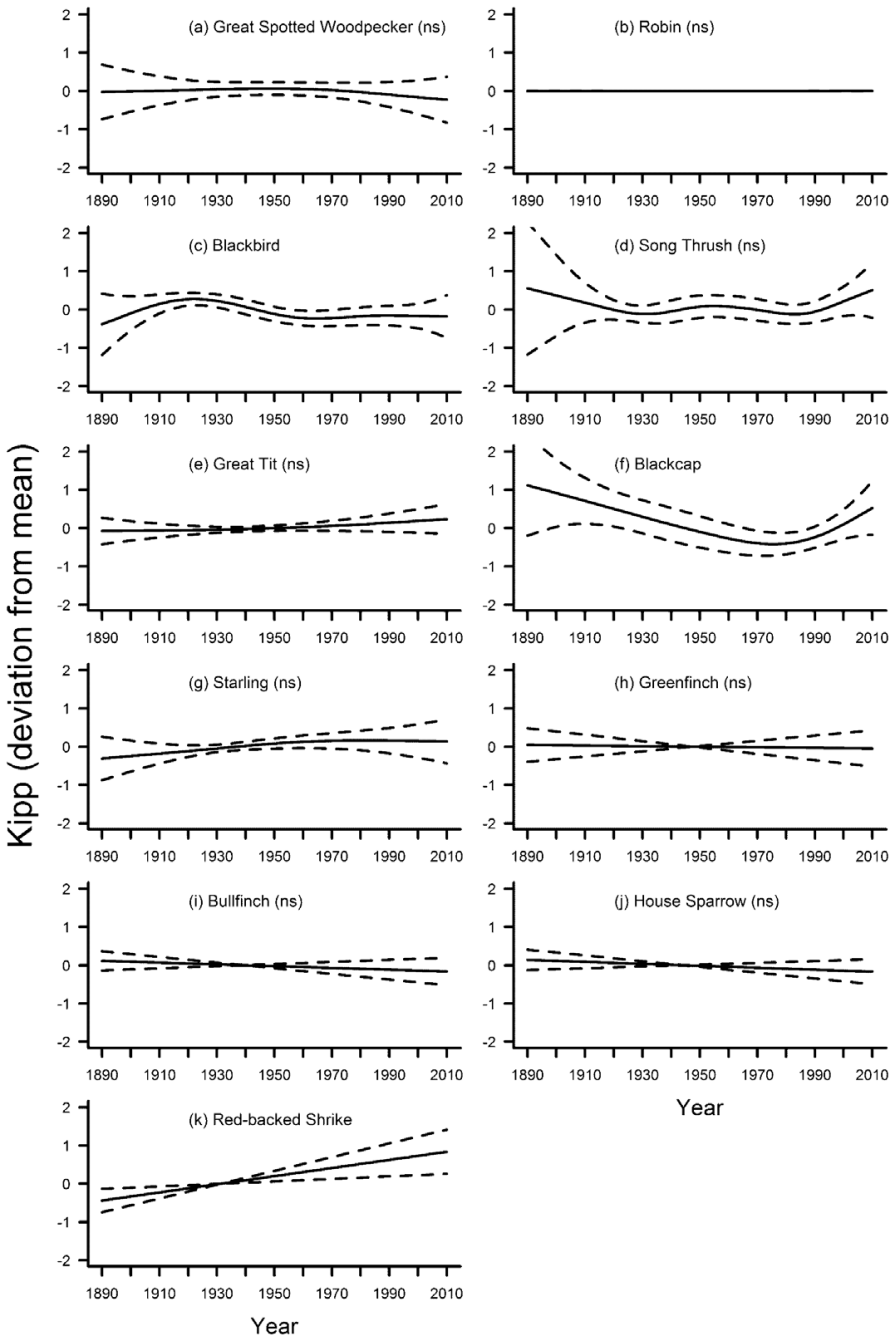
Fluctuations in wing pointedness (Kipp) of eleven bird species in southern Germany between 1889 and 2010. Solid line – regression spline fit from the GAM; dashed lines – 95% confidence intervals. See methods for details. A p-value of >0.05 is indicated with (ns) after the species name, see table 2 for exact p-values.

**Figure 7:**
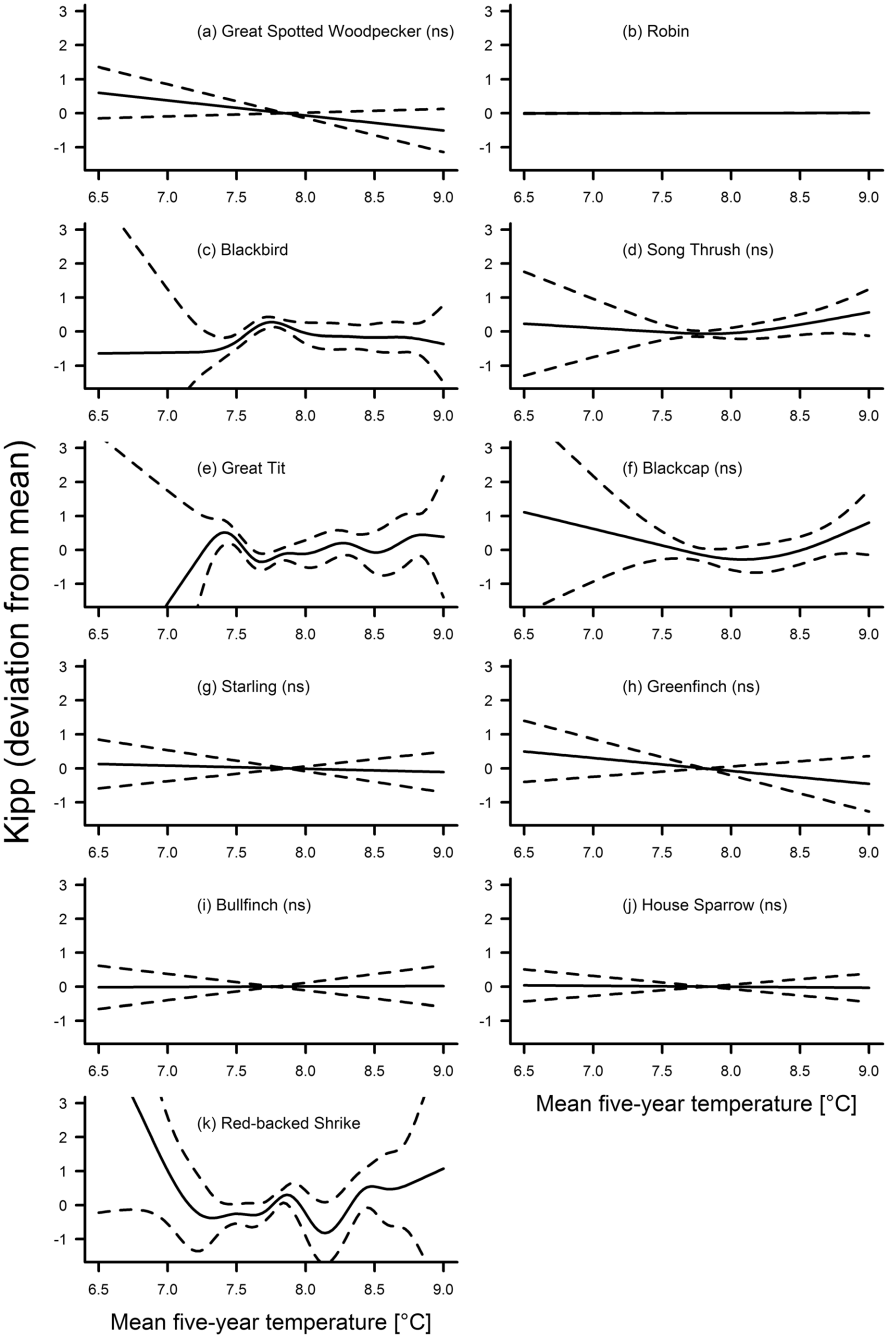
Fluctuations in wing pointedness (Kipp) of eleven bird species in association with the five-year mean annual temperature. Solid line – regression spline fit from the GAM; dashed lines – 95% confidence intervals. See methods for details. A p-value of >0.05 is indicated with (ns) after the species name, see table 3 for exact p-values.

Variation in Kipp was significantly associated with the smooth term year in only three species (Table 2). Kipp declined in the blackbird from the 1920s to the 1960s, after which it was constant (Figure 6c). In the blackcap, it showed a constant decrease until about 1980, after which it increased again (Figure 6f). In the red-backed shrike, Kipp increased linearly throughout the study period (Figure 6k). In four species, Kipp was significantly associated with the five-year mean of temperatures (Table 3), but there was no particular trend in any of these species (Figure 7). In the blackbird, the great tit and the red-backed shrike, the GAM including temperature as a smooth term explained significantly more variation of the data than the GAM including year as the smooth term. In the robin, both models explained a similar amount of variation of the data (Table 4).

## Discussion

Our study found no support for the hypothesis of a general decrease in surrogates for body size in times of global warming, and no two species displayed similar changes in morphology over time when all three morphological traits were considered together. In the majority of species, there was no consistent variation in tarsus length across the study period (Figure 2), but starling, greenfinch and house sparrow showed remarkable parallel fluctuations. Wing length varied significantly and mostly decreased for seven out of eleven species (Figure 5). There was a non-significant linear decrease in two additional species, and in one species the non-significant fluctuations included a distinct decrease in wing length during the last decades (Figure 5). Kipp showed in general no significant fluctuations throughout the study period (Figure 6). Hardly any of the morphological traits was significantly associated with temperature. When they were, models including year as the smooth term often explained significantly more variation in the data. Overall, our results indicate that: (1) independent patterns of long-term variation in multiple morphological traits have occurred within individual species; and (2) species-specific patterns of long-term variation for each trait have been the rule rather than the exception.

The lack of support for the general hypothesis that birds have become smaller during periods of global warming is in contrast to other studies that claimed a recent general decrease in body size of birds [12], [17], [63], [64] (but see [20], [22]). Many of these studies used wing length as a surrogate for body size which may not be an appropriate predictor of overall body size [39]–[41]. In our study, wing lengths also declined in a number of species throughout the study period, but not in correlation with temperature. Wing length is also related to migratory activity with shorter wings indicating shorter migration distances [36], [37]. The mean migration distance of many bird populations, as well as the proportion of migratory individuals within populations, have decreased in recent decades presumably due to climate change [47], [65]. This may have released populations from the selective pressure to optimise wing shape for migration. Thus, a reduction in wing length may reflect decreasing migratory activity as a response to climate change, rather than decreasing body size. However, a decrease in migration distances or the proportion of migratory individuals should also lead to a reduction in wing pointedness [36], [37], [46]. We found evidence of reduced wing pointedness only in the blackbird, a species for which a reduction in migratory activity has been shown [66], and only during a limited period. Thus, the hypothesis that decreasing wing length is a response to the reduction of migratory activity remains to be validated and the observed changes may have been caused by other ecological factors such as change in habitat availability [67]. However, decreasing wing length is unlikely to indicate a reduction in body size as a response to increased temperature.

Choice of morphological characters determines patterns of change over time [68]. Tarsus length has been considered to be a more appropriate surrogate for size than wing length [39]–[41]. In our study, tarsus length did not show any consistent trend among species. Where there were significant fluctuations in tarsus length over time or with temperature, they did not necessarily support the a priori hypothesis of a decreasing trend. Indeed, four species (Figure 2d,g,h,j) showed a pronounced increasing trend during the recent decades of accelerated global warming. Nestlings of birds grow larger tarsi when reared under more favourable conditions [61], [69]. Therefore, better foraging conditions related to higher temperatures may result in larger tarsi, yielding the opposite trend to the predicted adaptive change to varying thermoregulatory needs (see also [20]).

Treating wing and tarsus length as surrogates for body size leads to some contradictory results (e.g. trends during the last decades for great-spotted woodpecker, Figure 2a, 5a; starling, Fig. 2g, 5g; house sparrow, Figure 2j, 5j). Similar contradictions have also been found in previous studies [70], further indicating pitfalls when interpreting trends in single measurements. The conclusion that body size in birds has declined in response to climate change may have been biased in some studies by the use of wing length as the only measure for size.

The low support for models that included temperature as the smoothing term also suggests that climate change has not been a universally important driver of morphological change. Using long term data sets of museum specimens, change in bill length in the Hawaiian i'iwi *Vestiaria coccinea* was associated with altered food sources [27]. An increase in wing pointedness in North American forest passerines was linked to habitat fragmentation [67]. House sparrows in urbanised habitats have shorter tarsi than those living in rural areas [71]. Changes in prey availability have been suggested as the cause of variation in size of the European goshawk *Accipiter gentilis* [28], [72]. Therefore there are many hints that various factors influence morphology irrespective of climate change or only indirectly related to it.

In conclusion, we found almost no morphological variation that was directly correlated with change in five-year means of temperatures. The general warming that has occurred in southern Germany since the 1880s was not paralleled by the expected change in surrogates for birds' body size. We argue that use of long-term datasets and non-linear models of change over time, along with consideration of multiple surrogates, may prompt similar conclusions in other regions or globally. Furthermore, drivers of adaptation other than ambient temperature [73], [74] should be considered when trying to understand fluctuations in morphology.

## Supporting Information

Figure S1 **Result of the repeated measurement of 95 tarsus lengths of blackbirds.** Shown are the differences between the two measurements of a single bird (dots) and the result of a linear regression of the differences between the two measurements (d Measurement) on the year of collection of the respective individuals (F_1, 93_<0.001, p = 0.988, adjusted R^2^<0.001). The regression line is shown and it is virtually identical with a line with the function y = 0 which would also be the result of such a regression when there would be no differences between the repeated measurements at all. Therefore our measurements can be used to analyse size trends despite a relatively low repeatability between measurements.
(DOC)

Table S1 **Numbers of specimens considered per year and species.**
(DOC)
