# Supplementary material for: Morphological Change to Birds over 120 Years Is Not Explained by Thermal Adaptation to Climate Change
Source: PLoS One. 2014 Jul 14;9(7):e101927. doi: 10.1371/journal.pone.0101927 (PMC4096916; doi:10.1371/journal.pone.0101927)
Supplement: Figure S1 — Result of the repeated measurement of 95 tarsus lengths of blackbirds. Shown are the differences between the two measurements of a single bird (dots) and the result of a linear regression of the differences between the two measurements (d Measurement) on the year of collection of the respective individuals (F1, 93<0.001, p = 0.988, adjusted R2<0.001). The regression line is shown and it is virtually identical with a line with the function y = 0 which would also be the result of such a regression when there would be no differences between the repeated measurements at all. Therefore our measurements can be used to analyse size trends despite a relatively low repeatability between measurements. (DOC) [file pone.0101927.s001.doc]

**Figure S1.** **Result of the repeated measurement of 95 tarsus lengths of blackbirds.** Shown are the differences between the two measurements of a single bird (dots) and the result of a linear regression of the differences between the two measurements (d Measurement) on the year of collection of the respective individuals (F_1, 93_ < 0.001, p = 0.988, adjusted R² < 0.001). The regression line is shown and it is virtually identical with a line with the function y = 0 which would also be the result of such a regression when there would be no differences between the repeated measurements at all. Therefore our measurements can be used to analyse size trends despite a relatively low repeatability between measurements.

**
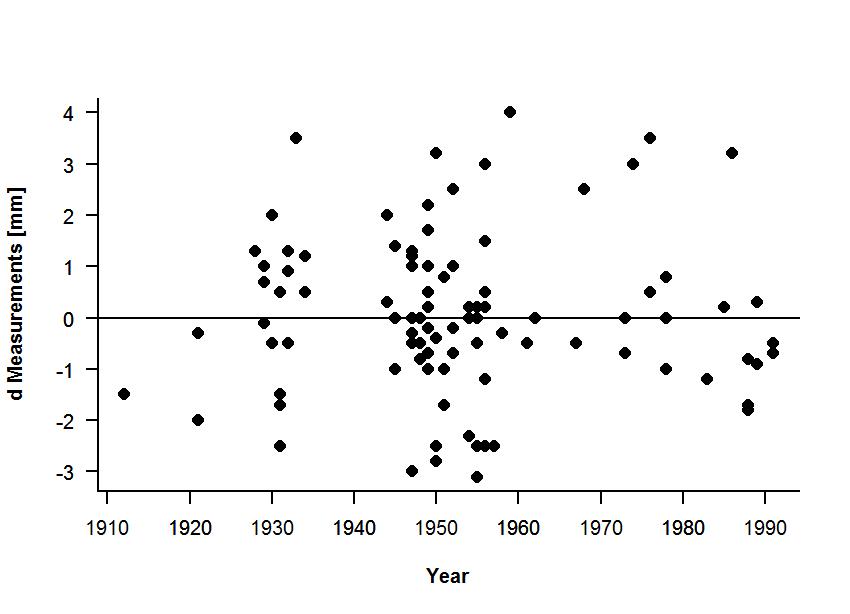
**
