# Supplementary material for: Morphological Change to Birds over 120 Years Is Not Explained by Thermal Adaptation to Climate Change
Source: PLoS One. 2014 Jul 14;9(7):e101927. doi: 10.1371/journal.pone.0101927 (PMC4096916; doi:10.1371/journal.pone.0101927)
Supplement: Table S1 — Numbers of specimens considered per year and species. (DOC) [file pone.0101927.s002.doc]

**Table S1.** Numbers of specimens considered per year and species.

| **Year** | **Great Spotted Woodpecker** | **European Robin** | **Common Blackbird** | **Song Thrush** | **Great Tit** | **Eurasian Blackcap** | **Common Starling** | **Greenfinch** | **Bullfinch** | **House sparrow** | **Red-backed Shrike** |
| --- | --- | --- | --- | --- | --- | --- | --- | --- | --- | --- | --- |
| 1889 |  |  |  |  |  |  |  |  |  | 2 |  |
| 1890 |  |  |  |  |  |  |  |  |  | 2 |  |
| 1892 |  |  |  |  |  |  |  |  |  |  | 1 |
| 1893 | 1 |  |  |  |  |  |  |  |  |  |  |
| 1894 |  | 1 | 2 |  |  |  |  |  |  | 2 |  |
| 1895 |  | 3 | 2 |  | 2 | 2 |  | 1 |  | 1 | 3 |
| 1896 | 2 | 1 |  |  |  |  |  |  |  |  | 1 |
| 1897 | 2 | 1 | 6 |  | 4 |  |  |  |  | 9 |  |
| 1898 | 1 |  |  |  | 2 | 2 |  | 3 |  |  | 7 |
| 1899 |  |  |  |  | 3 | 3 |  | 6 |  | 2 | 7 |
| 1900 |  |  |  | 1 |  |  | 1 | 1 |  |  |  |
| 1901 |  |  | 1 |  |  |  |  | 1 |  |  |  |
| 1902 | 1 |  |  |  |  |  | 2 | 3 |  | 1 | 6 |
| 1903 |  | 4 | 1 | 1 | 1 |  | 1 |  |  |  | 1 |
| 1904 |  |  | 2 |  | 1 |  | 1 |  |  | 3 |  |
| 1905 |  | 2 | 1 |  | 2 |  | 3 | 5 |  |  |  |
| 1906 | 1 | 2 | 3 |  | 2 |  | 11 | 2 | 1 | 5 | 3 |
| 1907 |  |  | 1 |  |  |  |  |  | 1 | 3 | 1 |
| 1908 |  |  | 4 |  | 10 |  |  |  | 6 | 1 | 2 |
| 1909 | 1 | 1 | 3 | 1 | 3 | 1 | 1 | 5 | 2 | 6 | 10 |
| 1910 | 9 | 4 | 7 |  | 6 |  | 1 | 2 | 10 | 5 | 7 |
| 1911 | 4 | 2 | 4 | 2 | 9 |  | 9 | 18 | 11 | 11 | 3 |
| 1912 | 5 | 3 | 8 | 2 | 8 | 1 | 2 | 13 |  | 16 | 1 |
| 1913 | 1 | 2 | 9 | 5 | 4 | 1 | 3 | 7 | 2 | 5 |  |
| 1914 | 1 | 2 | 10 | 1 | 10 | 2 | 15 | 6 | 6 | 3 | 2 |
| 1915 | 1 | 2 | 5 | 2 | 4 |  | 8 | 3 | 2 | 3 | 1 |
| 1916 |  | 6 | 12 |  | 8 | 2 | 5 | 3 | 12 |  | 9 |
| 1917 | 1 | 7 | 3 | 2 | 8 | 2 | 9 | 10 | 18 | 2 | 10 |
| 1918 | 4 | 1 | 3 | 2 |  | 3 | 5 | 2 | 4 | 3 |  |
| 1919 | 8 | 1 | 11 | 6 | 6 |  | 1 | 4 | 23 |  | 9 |
| 1920 | 6 | 1 | 5 | 3 | 7 | 1 | 5 | 7 | 39 | 8 | 1 |
| 1921 | 1 | 2 | 7 |  | 13 | 1 | 6 | 3 | 53 | 1 | 3 |
| 1922 | 3 | 16 | 6 | 19 | 7 |  | 13 | 2 | 9 |  | 6 |
| 1923 | 6 | 7 | 6 | 4 | 5 | 1 | 22 | 9 | 5 | 11 | 5 |
| 1924 | 5 | 10 | 19 | 5 | 8 | 5 | 17 | 5 | 7 | 13 | 8 |
| 1925 | 15 | 6 | 19 | 5 | 14 |  | 8 | 8 | 4 | 5 | 10 |
| 1926 | 8 | 7 | 5 |  |  | 1 | 13 | 7 | 2 | 13 | 4 |
| 1927 | 8 | 4 | 10 | 4 | 5 | 1 | 12 | 8 | 8 | 3 | 5 |
| 1928 |  | 1 | 4 | 5 |  | 2 | 6 | 12 | 10 | 1 | 6 |
| 1929 | 1 | 11 | 7 | 5 | 9 | 1 | 23 | 33 | 1 | 13 | 21 |
| 1930 | 8 | 15 | 27 | 9 | 13 | 5 | 14 | 5 | 7 | 43 | 10 |
| 1931 | 6 | 18 | 7 | 7 | 11 | 1 | 12 | 7 | 4 | 47 | 13 |
| 1932 | 1 | 3 | 5 | 12 | 6 | 1 | 7 | 10 | 1 | 8 | 6 |
| 1933 | 3 | 2 | 3 | 8 | 3 | 1 | 2 | 3 | 18 | 3 | 5 |
| 1934 | 4 | 4 | 3 | 4 | 6 |  | 3 | 4 | 16 | 4 | 3 |
| 1935 | 3 | 1 | 1 | 4 | 3 |  | 2 | 1 | 2 | 7 | 1 |
| 1936 | 1 |  | 3 | 2 | 2 |  | 2 | 1 | 2 | 5 | 1 |
| 1937 | 3 |  | 4 | 1 | 3 | 1 | 9 | 2 | 3 | 27 |  |
| 1938 |  | 4 | 3 | 1 | 5 |  | 2 | 5 | 3 | 2 | 1 |
| 1939 | 1 | 1 | 3 | 2 | 1 | 2 | 1 | 3 | 1 | 10 |  |
| 1940 | 1 |  |  | 1 |  |  | 3 |  | 1 |  |  |
| 1941 | 1 |  |  | 1 |  | 1 |  | 1 | 1 |  |  |
| 1942 | 1 |  | 0 |  |  |  |  | 1 |  |  |  |
| 1943 |  |  |  |  |  |  | 1 |  |  |  |  |
| 1944 | 1 |  | 3 | 2 | 3 | 1 | 2 | 3 |  | 7 |  |
| 1945 | 1 | 2 | 5 |  | 10 |  | 3 | 13 | 12 | 9 |  |
| 1946 | 1 | 2 |  |  | 12 | 1 | 6 | 35 | 11 | 38 | 3 |
| 1947 | 3 | 3 | 13 | 3 | 18 | 2 | 2 | 39 | 3 | 50 | 1 |
| 1948 | 4 | 6 | 10 | 3 | 26 |  | 11 | 1 | 1 | 7 | 1 |
| 1949 |  | 4 | 10 | 2 | 4 |  | 2 | 6 | 3 | 7 | 2 |
| 1950 | 3 | 8 | 4 | 2 | 5 | 2 | 13 | 4 | 8 | 16 | 6 |
| 1951 | 3 | 1 | 9 | 5 | 11 | 7 | 5 | 1 | 4 | 11 | 4 |
| 1952 | 1 | 5 | 5 | 6 | 10 | 1 | 2 | 3 | 5 | 28 | 3 |
| 1953 | 6 | 4 | 3 | 2 | 2 | 2 | 3 | 7 | 4 | 6 | 4 |
| 1954 | 3 | 7 | 8 | 3 | 3 | 2 | 6 | 10 | 6 | 4 | 3 |
| 1955 | 5 | 1 | 6 | 2 | 2 | 1 | 2 | 2 | 1 | 9 | 4 |
| 1956 | 2 | 2 | 8 | 3 | 1 |  |  | 3 | 2 | 10 | 2 |
| 1957 | 9 |  | 5 | 2 | 4 | 1 | 1 | 1 | 2 | 3 | 4 |
| 1958 | 2 | 4 | 3 | 6 | 3 | 1 | 5 | 5 | 3 | 7 | 1 |
| 1959 | 3 | 3 | 3 | 5 | 5 | 3 | 4 | 4 | 10 | 5 | 2 |
| 1960 | 3 | 2 | 5 | 3 | 1 | 1 | 8 | 4 | 3 | 2 | 2 |
| 1961 | 3 | 1 | 4 | 5 | 7 | 3 | 11 | 11 | 6 | 2 | 1 |
| 1962 | 4 | 2 | 4 |  |  | 1 | 3 | 5 | 2 | 1 | 3 |
| 1963 |  | 2 | 3 | 1 | 2 | 1 | 1 | 1 | 7 | 2 | 1 |
| 1964 | 2 | 4 | 5 | 3 | 7 | 2 |  | 9 | 12 | 6 | 1 |
| 1965 |  | 1 | 4 |  | 3 |  | 3 | 4 | 2 | 1 |  |
| 1966 | 4 | 5 | 7 | 2 | 4 | 4 | 1 | 3 | 4 | 11 | 3 |
| 1967 | 4 | 4 | 7 | 3 | 7 | 1 | 3 | 5 | 6 | 5 | 1 |
| 1968 |  | 2 | 4 | 1 | 5 |  | 3 | 1 | 6 | 1 |  |
| 1969 | 3 | 3 | 4 | 2 | 2 | 1 | 3 | 1 | 4 | 2 | 1 |
| 1970 | 3 | 6 | 5 |  |  | 2 | 1 | 5 | 4 | 3 |  |
| 1971 | 4 |  | 7 | 1 | 2 | 1 |  | 9 | 2 | 3 | 1 |
| 1972 | 7 | 1 | 4 | 4 | 5 | 2 | 2 | 7 | 4 | 5 | 1 |
| 1973 | 5 | 4 | 6 | 3 | 7 | 5 | 1 | 3 | 6 | 10 |  |
| 1974 | 1 | 2 | 4 | 2 | 4 | 2 | 2 | 4 | 1 | 9 | 1 |
| 1975 | 3 | 4 | 3 | 7 | 7 | 4 |  | 8 | 8 | 1 | 2 |
| 1976 | 4 | 3 | 3 | 2 | 5 | 6 |  | 2 | 1 | 3 |  |
| 1977 | 3 | 5 | 2 | 1 | 1 | 6 | 2 | 2 | 2 | 16 |  |
| 1978 | 3 | 3 | 3 | 2 | 6 | 3 | 1 | 1 | 2 | 3 | 1 |
| 1979 | 7 | 4 | 6 | 4 | 2 | 1 | 6 | 2 | 3 | 8 |  |
| 1980 | 3 | 7 | 5 | 2 | 5 | 4 | 2 | 1 | 1 | 2 | 2 |
| 1981 |  | 7 | 1 |  | 2 | 6 | 2 | 4 | 3 | 3 |  |
| 1982 | 2 | 1 | 1 | 4 |  | 1 |  | 3 | 2 | 1 |  |
| 1983 | 3 | 6 | 2 | 4 | 3 | 6 | 2 | 4 |  | 4 |  |
| 1984 | 3 | 5 | 7 | 2 | 3 | 3 |  | 2 | 2 | 4 |  |
| 1985 | 3 | 3 | 1 | 10 | 2 | 4 | 2 | 2 | 7 | 2 |  |
| 1986 | 4 | 2 | 5 | 4 | 4 | 4 | 3 | 2 | 3 |  | 1 |
| 1987 | 5 | 9 | 3 | 2 | 1 | 6 | 1 | 2 | 5 | 2 |  |
| 1988 | 3 | 11 | 4 | 4 | 3 | 9 | 1 | 3 | 7 | 2 | 1 |
| 1989 | 4 | 2 | 6 | 10 | 1 | 1 | 2 | 4 | 4 | 2 | 1 |
| 1990 | 7 | 5 | 1 | 2 | 1 | 1 | 4 | 7 | 4 | 3 |  |
| 1991 | 4 | 10 | 5 | 1 |  | 3 |  | 4 | 4 | 1 | 2 |
| 1992 | 2 | 1 | 1 | 1 |  | 2 | 1 | 2 | 3 | 7 |  |
| 1993 | 1 | 5 | 2 | 1 | 3 | 2 |  | 1 |  | 3 |  |
| 1994 | 2 |  | 5 | 2 |  | 2 | 4 | 3 | 1 |  | 1 |
| 1995 | 1 |  | 3 |  |  | 1 | 2 |  |  | 1 | 1 |
| 1996 | 6 | 2 |  | 2 | 1 | 4 |  | 1 |  |  |  |
| 1997 | 1 | 1 | 2 | 1 | 2 |  | 3 | 1 | 1 | 3 |  |
| 1998 | 2 | 2 | 1 |  |  | 3 | 2 | 4 | 2 | 2 |  |
| 1999 | 3 | 2 |  | 3 | 1 |  |  |  |  | 1 | 1 |
| 2000 | 1 | 5 | 1 | 2 | 1 |  |  | 1 |  |  |  |
| 2001 | 2 | 6 | 6 | 1 | 1 |  | 1 |  |  | 1 | 1 |
| 2002 | 5 |  | 3 | 1 |  |  |  | 1 | 1 | 1 |  |
| 2003 | 3 | 1 | 1 |  | 1 | 1 |  |  |  | 2 |  |
| 2004 | 4 | 1 | 1 | 3 | 1 |  | 3 | 1 | 3 | 1 |  |
| 2005 | 1 | 2 | 5 | 1 | 6 | 3 |  | 1 | 1 | 1 |  |
| 2006 |  | 1 | 3 |  | 2 | 4 |  | 1 |  |  |  |
| 2007 | 1 | 1 | 3 | 4 | 2 | 3 | 2 |  |  | 1 |  |
| 2008 | 3 | 7 | 5 | 4 |  | 3 | 1 | 1 | 1 |  | 1 |
| 2009 |  | 4 | 2 |  | 3 | 1 |  | 1 |  | 1 |  |
| 2010 |  |  |  |  |  | 1 | 2 | 1 |  |  |  |
| **Total** | **318** | **377** | **515** | **290** | **459** | **192** | **419** | **514** | **510** | **671** | **264** |
